# Supplementary material for: Differentiating Dyes: A Spectroscopic Investigation into the Composition of Scarlet Bloodroot (Haemodorum coccineum R.Br.) Rhizome
Source: Molecules. 2023 Nov 3;28(21):7422. doi: 10.3390/molecules28217422 (PMC10648959; doi:10.3390/molecules28217422)
Supplement: Supplementary file 1 [file molecules-28-07422-s001.zip › molecules-2666372-supplementary.pdf]

Supplementary Information

Differentiating Dyes: A spectroscopic investigation into the composition of Scarlet bloodroot (*Haemodorum coccineum* R.Br.) rhizome

Matheus Carpinelli de Jesus [1,*], Taylah Church [2], Greg Leach [2], David Leach [3], James J. De Voss [1] and Joanne T. Blanchfield [1,*]

[1] School of Chemistry and Molecular Biosciences, The university of Queensland, Brisbane, 4072, QLD, Australia
[2] Menzies School of Health Research, Darwin University, Darwin, NT, Australia
[3] Research and Development, Integria (MediHerb), Brisbane, QLD, Australia
* Correspondence: j.blanchfield@uq.edu.au; m.carpinellidejesus@uq.edu.au

Table of Contents

Scheme S1 Aromatic compounds used in the naming of phenylphenalenones isolated from plants in the *Haemodoraceae* family, with a color-coded naming scheme showing inconsistencies in naming present in current literature. Identified in boxes are the naming conventions used in this paper.

Figure S1 ^{1}H NMR (500 MHz, $CDCl_3$) for dilatrin (**12**)

Figure S2 ^{13}C NMR (500 MHz, $CDCl_3$) for dilatrin (**12**)

Figure S3 COSY (500 MHz, $CDCl_3$) for dilatrin (**12**)

Figure S4 HSQC (500 MHz, $CDCl_3$) for dilatrin (**12**)

Figure S5 HMBC (500 MHz, $CDCl_3$) for dilatrin (**12**)

Figure S6 MS (ESI) positive and negative modes for dilatrin (**12**)

Figure S7 ^{1}H NMR (500 MHz, $CDCl_3$) for **19**

Figure S8 HSQC (500 MHz, $CDCl_3$) for **19**

Figure S9 HMBC (500 MHz, $CDCl_3$) for **19**

Scheme S1 – Aromatic compounds used in the naming of phenylphenalenones isolated from plants in the *Haemodoraceae* family, with a color-coded naming scheme showing inconsistencies in naming present in current literature. Identified in boxes are the naming conventions used in this paper.

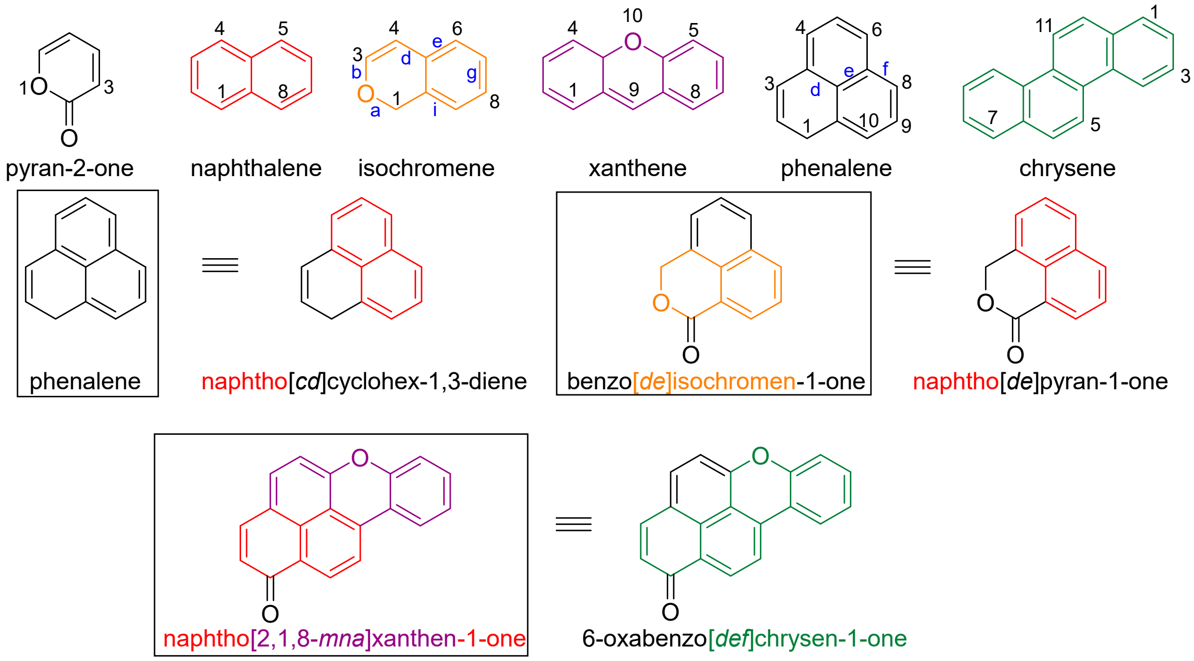

The naming of phenylphenalenones follows some interesting conventions. The cores receive compound names based on the smaller units which form the structure. Some common compounds used in the naming are represented in Scheme S1, with their carbon numbering and bond lettering. Some inconsistencies arise from the arbitrary use of smaller molecules, as demonstrated in Scheme S1. For consistency throughout, elucidation and naming will follow that used in the structures inside the box in Scheme S1. In cases where literature has used the other naming scheme, or the compounds has a common name, these will be stated as well.

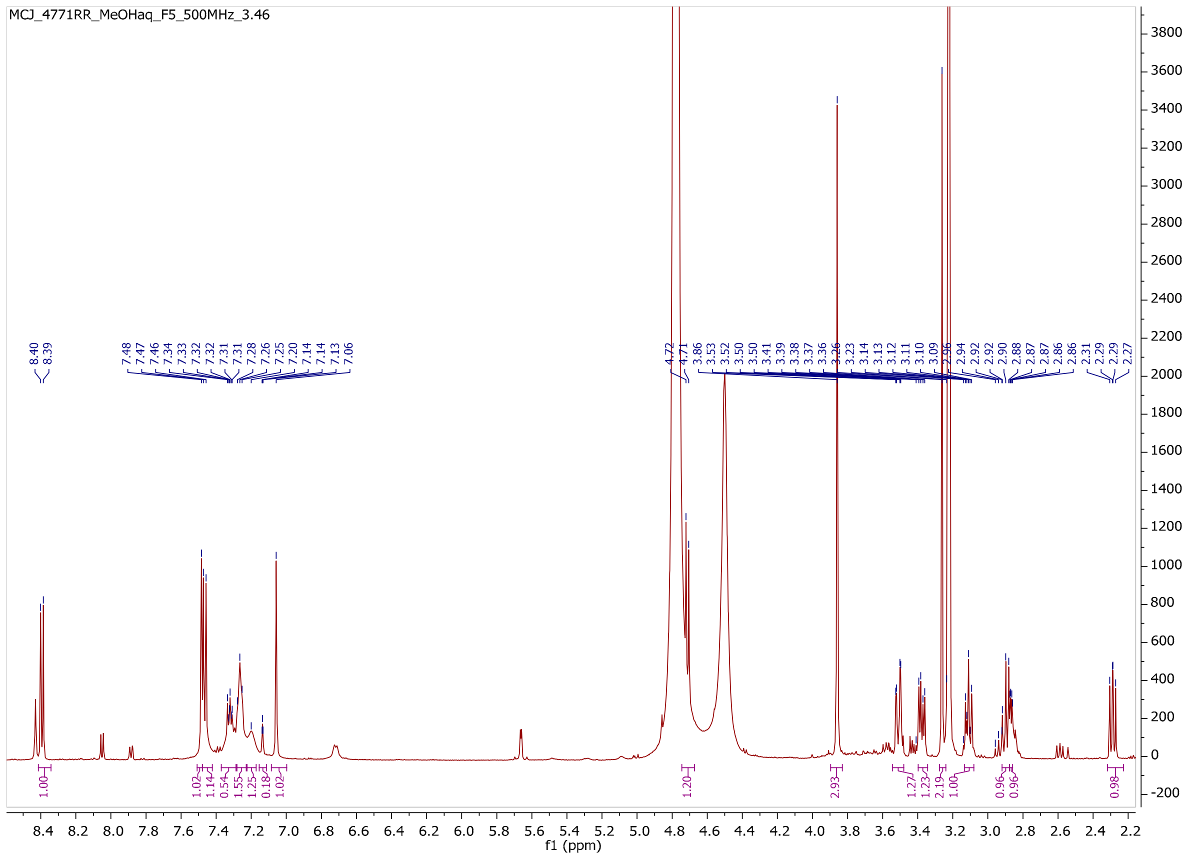

Figure S1 - ^{1}H NMR (500 MHz, $CDCl_3$) for dilatrin (**12**)

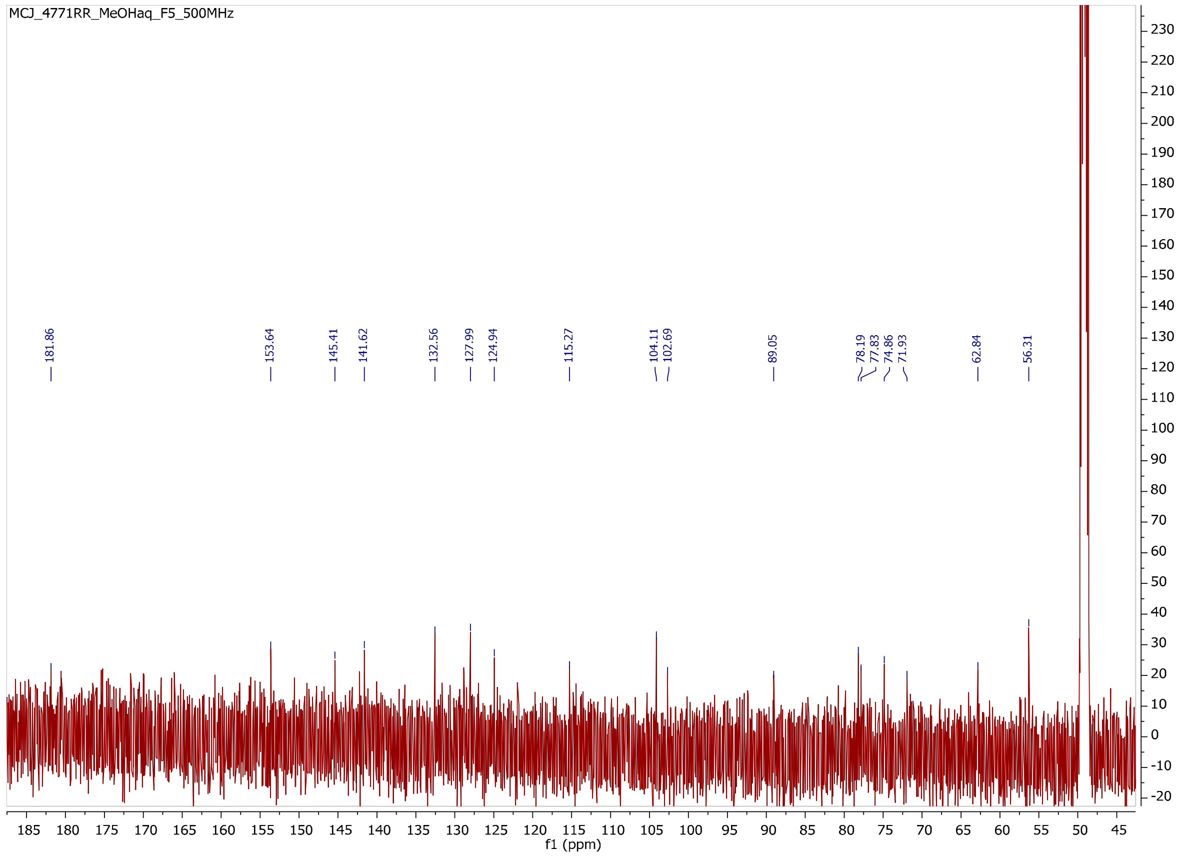

Figure S2 - ^{13}C NMR (500 MHz, $CDCl_3$) for dilatrin (**12**)

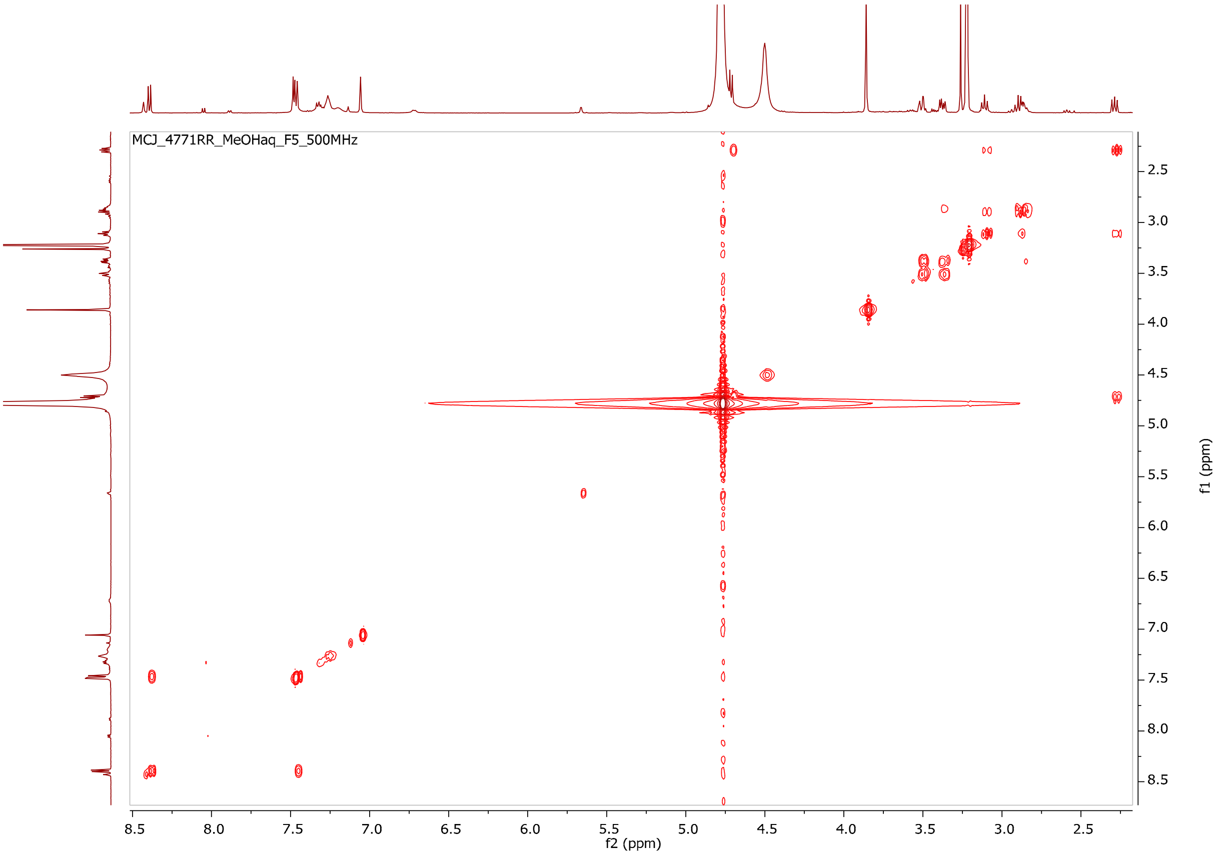

Figure S3 - COSY (500 MHz, $CDCl_3$) for dilatrin (**12**)

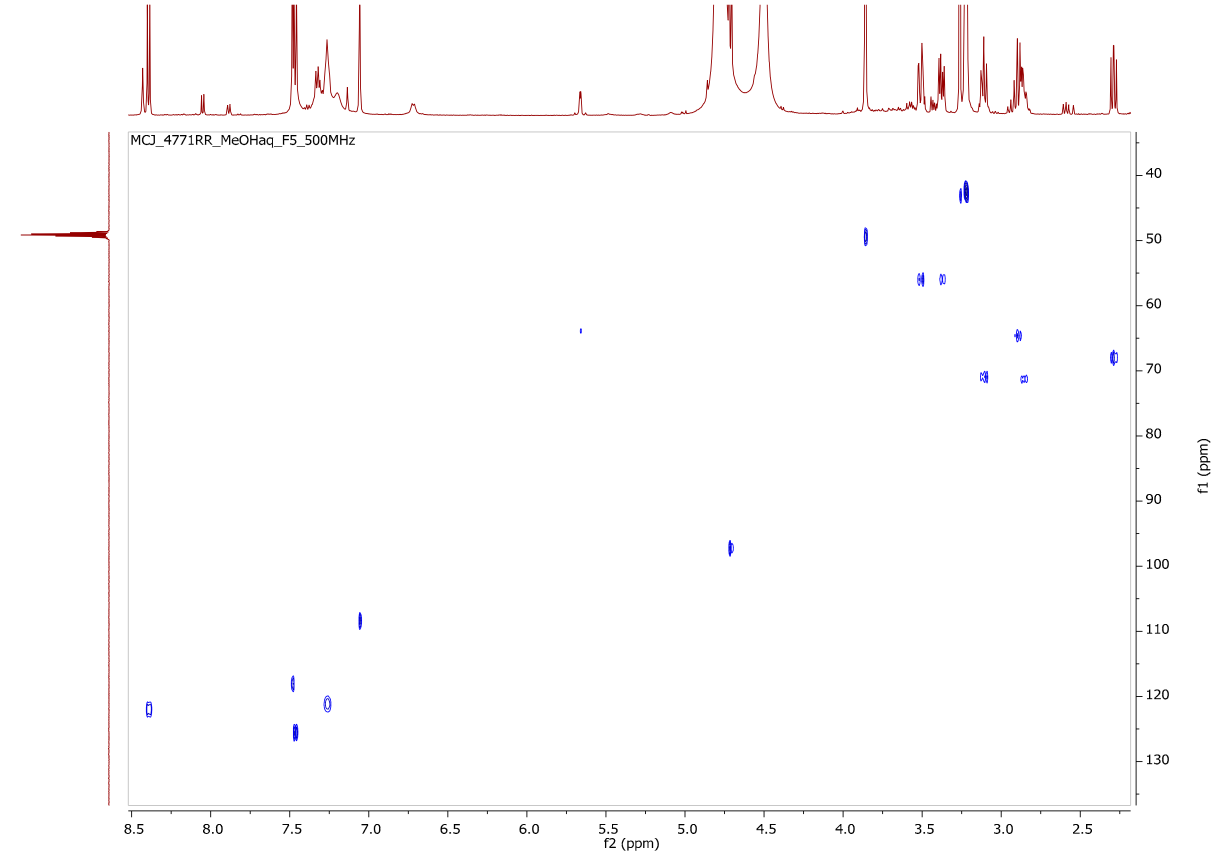

Figure S4 - HSQC (500 MHz, $CDCl_3$) for dilatrin (**12**)

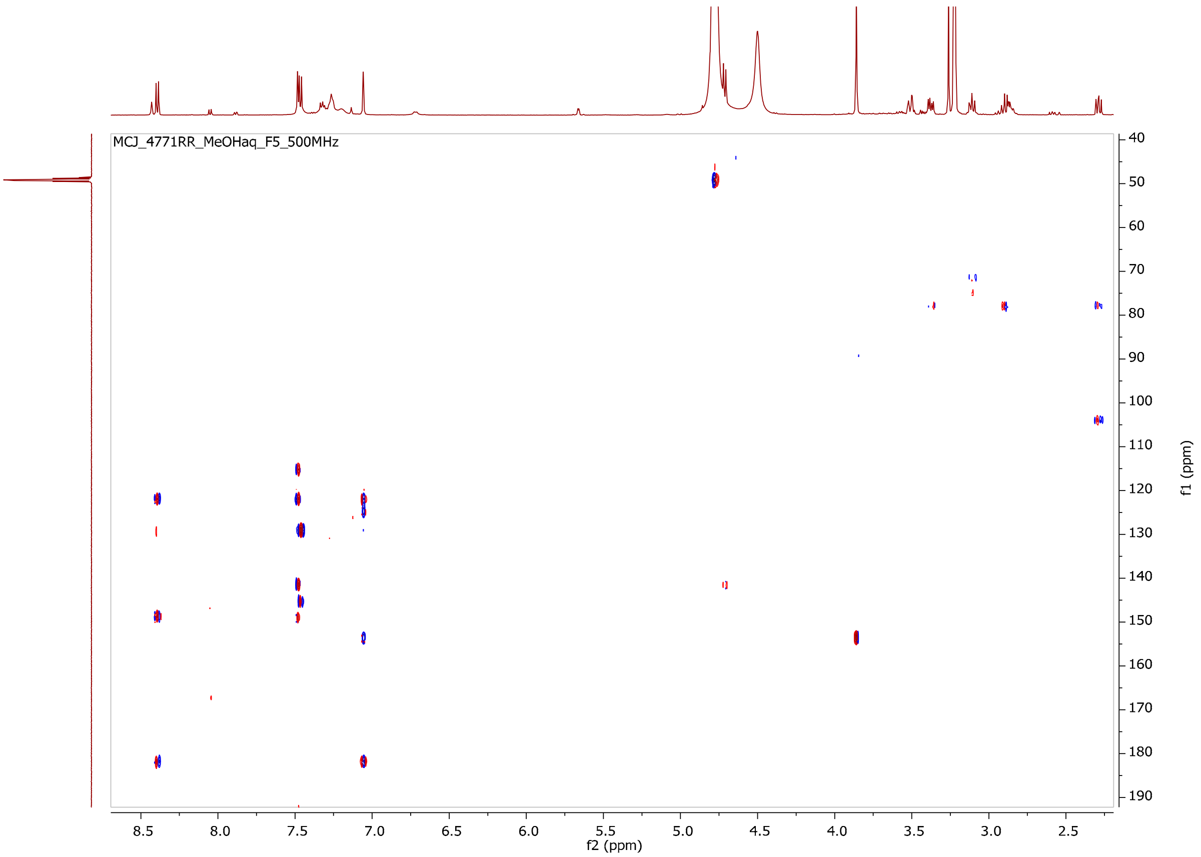

Figure S5 - HMBC (500 MHz, $CDCl_3$) for dilatrin (**12**)

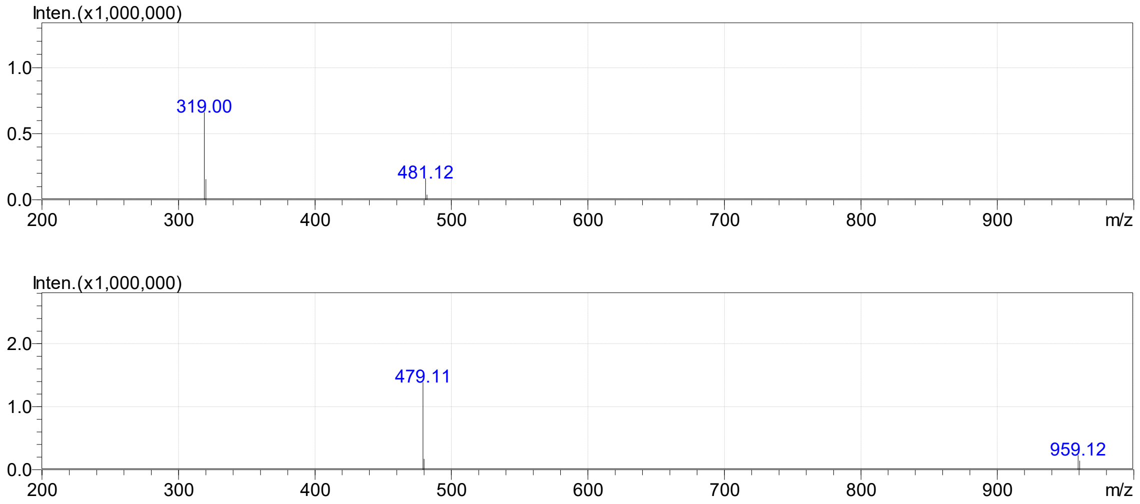

Figure S6 - MS (ESI) positive (top) and negative (bottom) modes for dilatrin (**12**)

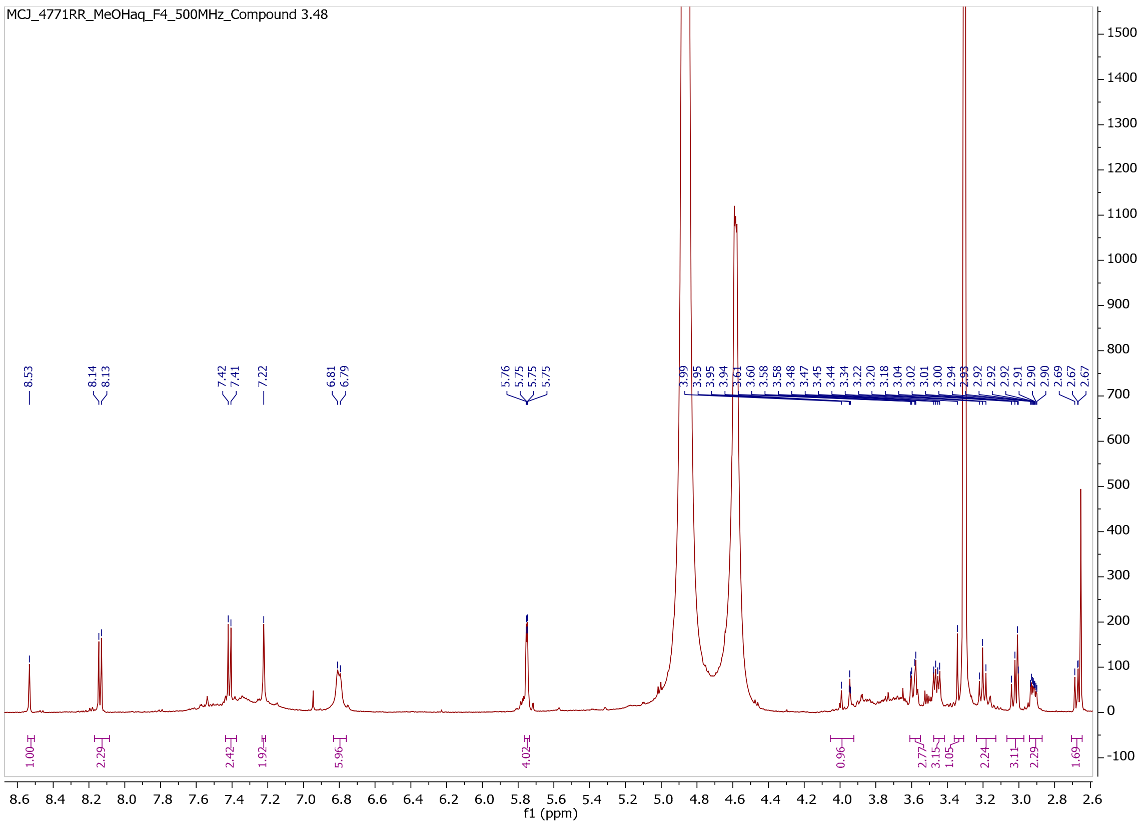

Figure S7 - ^{1}H NMR (500 MHz, $CDCl_3$) for **19**

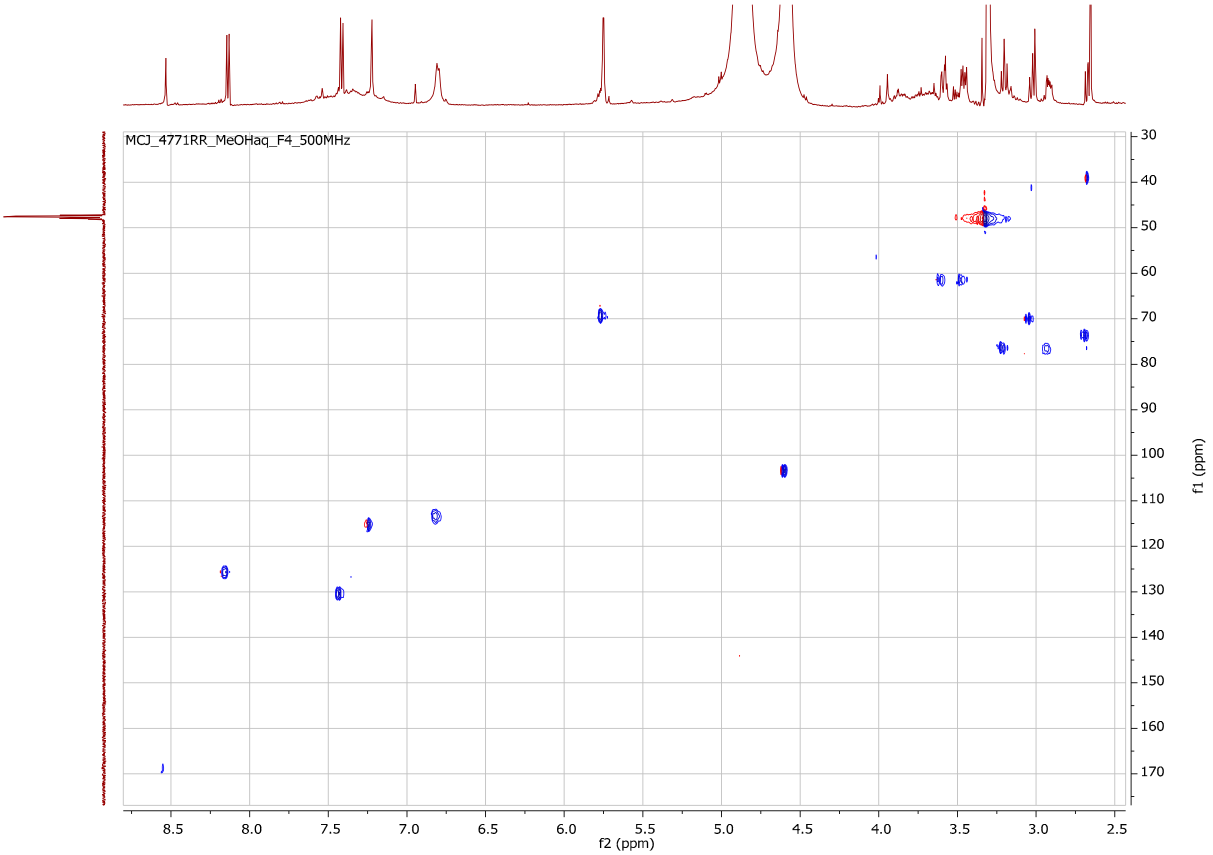

Figure S8 - HSQC (500 MHz, $CDCl_3$) for **19**

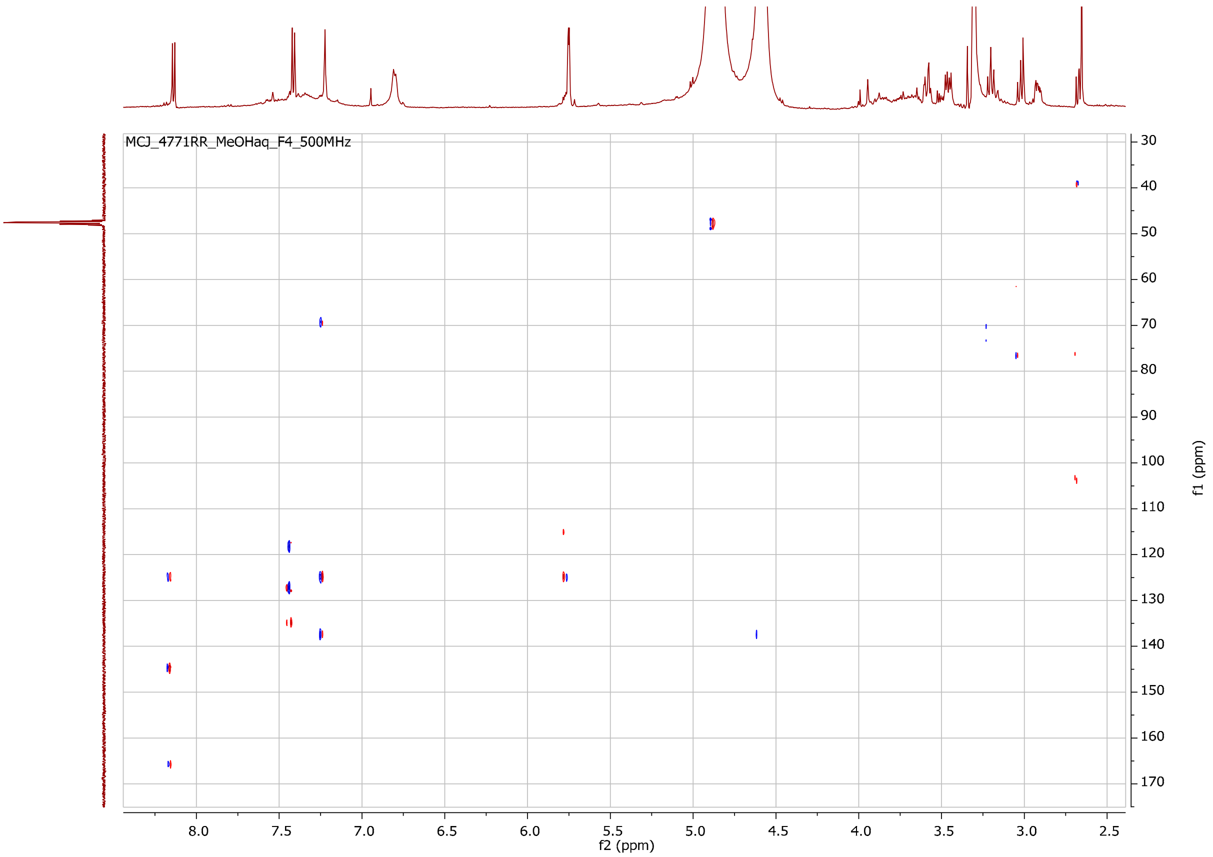

Figure S9 - HMBC (500 MHz, $CDCl_3$) for **19**
